# Slow and Secret Poisoning

**Published:** 1862-04

**Authors:** 


### Art. IV.—SLOW AND SECRET POISONING.

Talk not to us of *ghosts*. Commend us rather, for its tragic power, to a well-concocted story of slow and secret poisoning in the upper ranks of life; in a palace, if you will; or, better still, let the victim be a royal personage, and the murderer an incarnate fiend, who shall be proved, at the end of the story, to have been the death of half the people about the throne who have "paid the debt of nature" during the last quarter of a century. Let us gloat on the slow emaciation, the hollow eye, the sepulchral voice, the trembling limbs, the gradual decay of vigour both of body and mind; and at length on the miserable death of the victim on the very day predicted by a beautiful marchioness, herself the murderer! Such tales as these are the spice of history; who would read it without its proper mixture of romance? And what historian having thus successfully seasoned the dish, would not learn other ways of mingling the same nostrum, to be imitated by all newsmongers in all time coming! Besides being quite as full of romance and horror as the "true ghost-story," the "slow-poisoning" tale has an advantage over the latter; it is more readily believed by the mass of readers. There is scarcely enough of superstition now for a ghost story to *pay*. You rarely find one in a periodical; except, indeed, as a palpable joke, as in the instance in which the semi-transparent lady ghost entered a railway office, in order to telegraph to her husband that she was lying dead at the foot of a rock from which she had fallen. The general belief is, that ghosts are not real ghosts; that they have all been *laid* for half a century. On the other hand slow poisoning is believed to be possible, and the belief has found its way into the ranks of the profession. Moreover, it is a subject which of late has been a good deal mooted and canvassed and ventilated, and apparently even enacted and exemplified amongst us. We therefore think it is time that the profession, having been more than once caught tripping, should fairly face the question—*Is slow and secret poisoning possible?* Or, being possible, is it a likely thing? In other words, is it possible for a malicious wretch, who seeks to take away the life of another, so to mix with the victim's food or drink, without his being aware of it, such repeated doses of any poison as shall slowly and certainly destroy his life within a limited time, without the extreme probability of detection?

The first thing which would be requisite for the accomplishment of so diabolical a purpose would be the selection of a poison without colour, taste, or smell, or any other sensible property which might awaken the suspicion of the victim. It should not be capable of exciting vomiting, or purging, or griping, or ptyalism, or tetanic convulsions, or unnatural drowsiness; for any of these symptoms would awaken attention and inquiry. Neither should it be bulky, or very light, or insoluble in water, else it might easily be detected. But some of these objections apply to antimony, corrosive sublimate, strychnine, opium, and indeed, all the poisons with which we are at present acquainted, with the exception, perhaps, of arsenic. The white oxide of this metal (arsenious acid) is the poison which is generally supposed to have been used in the cases of slow and secret poisoning which have occupied the columns of our periodicals of late. It is without colour, smell, or taste: in small doses it neither nauseates, nor purges, nor gripes; it rarely excites ptyalism, never convulsions, nor drowsiness. It is moderately heavy, soluble in water, and poisonous in small quantities. Moreover, it is said to have the property of accumulating in the system when given in small doses, and then arresting the process of assimilation, and thus destroying life by slow degrees, but with certain effect. If all this be true, what more can a murderer desire? But *is* it true? Will arsenic destroy life slowly, insensibly, and with the probability that the cause will remain undetected? This question is full of interest, not only to the medical jurist, but to the whole community. For it appears we are in constant danger of this slow but certain death, not only from malicious foes, but from the artificial verdure wherewith ladies adorn their heads, not less than from the green papers which enliven our apartments;—not to mention the sufferings of the poor girls who, as artificial florists, are doomed, conspicuous in martyrdom, to lead off this "*dance of death.*" Nor is the opposite side of the question wholly destitute of interest; for it appears that while we unlucky English people hold life so loosely that "hundreds of young women and children" are annually sacrificed to the arsenic which is said to float in invisible insensible quantities in the atmosphere, the happier nymphs and swains of Styria are eating the poison as a luxury, in larger or smaller lumps, gaining plumpness beauty and vigour by its salutary action on the system, and at length depending upon it as a preservative of health, if not a necessary of life!

The most extraordinary fact connected with this subject is, that there are people who actually believe both of these accounts; at all events, there are periodicals which insist upon the truth of both;* nor does it seem to have occurred to any individual narrator that they cannot both be true! And we should be almost tempted to discuss this curious psychological fact, but that we have other business on hand.

It is not our purpose at present to investigate the comparative value of the testimony to these discordant statements, much less to attempt to reconcile them. We propose rather to inquire what is known of the effects of arsenic in different varieties of dose, particularly in such doses as are supposed to act on the human system slowly, secretly, and fatally. And the first point to which we beg the thoughtful attention of our readers is, that this question cannot be settled by mere popular testimony; for a judge and jury have tried their hands at it with the assistance of no common amount of medical testimony, and have signally failed! The case of supposed slow poisoning at Richmond must be fresh in the memory of all our readers. The jury found the cause of death to be the slow and secret operation of poison, given by the hand of a medical attendant, who was found guilty of the crime and sentenced to death. So clearly, however, did it afterwards appear that the lady might have died, not from arsenic or antimony

* See *Chambers's Journal*, part xcii., August, 1861.

(although traces of both were found in the tissues), but simply from long and continuous vomiting incident to pregnancy, that, on an appeal to the Crown, the supposed culprit was pardoned! The discrepancy of medical opinion saved his life.

This case conveys a lesson of caution to medical witnesses, not to offer a positive opinion on subjects not as yet scientifically investigated. And it ought to invest with interest some of the points discussed in this paper.

Let us inquire, then (as concisely as the subject will admit of), what is yet known of the action of arsenic on the human body. Arsenic may be taken (1) in large doses, either by accident or for the purpose of poisoning; or (2) in very minute doses for therapeutical purposes; or (3) in doses too small for rapid poisoning, and yet too large for medicinal purposes. We are tolerably well acquainted with its effects when from two or three grains to several drachms are swallowed in solution. It is almost invariably fatal, and generally within a few days or hours. Modern experience has also elicited very satisfactory evidence as to its physiological as well as therapeutical action in such minute doses as are found useful and safe in intermittents and in chronic cutaneous diseases. But with regard to its power of chronic poisoning in doses of an intermediate strength, our knowledge is as yet rather inferential and conjectural than experimental and positive.

Perhaps no writer has more carefully investigated the poisonous properties of arsenic in large doses, than Dr. Christison, of Edinburgh. In his elaborate work on poisons, he has so satisfactorily elucidated this subject as to make it almost superfluous to cite other authority. He has shown that arsenic has a twofold action: the one purely irritant, by virtue of which inflammation in the alimentary canal and elsewhere is rapidly produced; the other, a sympathetic or so-called narcotic action in parts or organs remote from the seat of its application. It is important to keep these distinctions in view, because this will throw light upon the ultimate subject of our inquiry.

Of all the arsenical compounds the arseniuretted-hydrogen, when inhaled, appears to be the most active, but this is never used for poisonous purposes; and our remarks will be chiefly confined to one preparation, the *white oxide*, or *arsenious acid*. How small a quantity of this familiar form of poison will, in one dose, occasion death? A medical witness should be very careful how he replies to such a question as this; for the effect depends not on the quantity swallowed, so much as on the quantity dissolved in the stomach: and not alone on this, for it is now an established fact that, in different individuals, there exists a greater variety of intolerance of arsenic than of any other poison.

It, therefore, never can be known with absolute certainty how small a quantity may chance to prove fatal in one case, or how large a quantity may be required to prove poisonous in another. Accordingly, we have cases recorded in which a dose of two grains has proved fatal, and others in which patients recovered after sixty grains, three-quarters of an ounce, and an ounce and a-half had been swallowed respectively. In these cases, however, the arsenic was taken immediately after a meal, followed by an emetic which speedily emptied the stomach of its contents.

*The symptoms of poisoning with large doses of arsenic* are by no means similar or uniform in every case. There is, however, most frequently a remarkable correspondence between the rapidity with which death follows the dose, and the prominent symptoms occurring in the case.

1. In cases in which *the patient dies in five or six hours or less* after swallowing the poison, the fatal symptoms will have been *excessive prostration of strength* and *frequent fainting*. And there may have been no other symptom; or, trivial vomiting and slight pain in the stomach *may* have occurred, and thus have suggested the idea of the source of the illness; but even when these mild gastric symptoms happen, they soon subside. There may also be some stupor or trifling convulsions preceding death; but the constant fainting, sometimes *ad deliquium*, is the leading symptom, and the practitioner is led rather to suspect internal hemorrhage than arsenical poisoning. These cases are rare, and they generally occur after very large doses of the poison have been swallowed, especially in solution. Here, death is produced not by inflammation, but by the shock undergone by the nervous system, or by a loss of power in the heart.

2. *When the patient survives from twenty-four hours to three days* (and such cases are the most frequent of all), the symptoms are very different. Sickness and faintness do indeed occur, and generally in the first instance (sometimes a few minutes after the arsenic is swallowed); but very soon afterwards the region of the stomach feels painful and commonly "burning," the pain being much aggravated by pressure. The vomiting and retching soon become violent, especially if drink is taken, and there is a sense of dryness, heat, and tightness in the throat, and generally intense thirst. Hoarseness and difficulty of speech commonly follow. The matter vomited is greenish or yellowish, and sometimes streaked or mixed with blood, particularly if the case lasts longer than a day.* Diarrhœa generally follows or (instead of it)

* Or it may be coloured by the soot or indigo now mixed with arsenic (as required by Act of Parliament), when sold in small quantities.

tenesmus; but in some cases the large intestines scarcely suffer at all. The pain in the stomach quickly becomes excruciating, and is often likened by the sufferer to a fire burning within him. The belly is tense and tender, sometimes swollen, but not generally. Now and then the whole alimentary canal is affected with burning pain from the throat to the anus, which is sore and excoriated. The mouth and lips have been sometimes observed to be inflamed, presenting dark specks or blisters. The irritation may also extend to the air-passages, and pneumonia may complicate the case. Other mucous membranes often suffer. Dysuria is very common, and the urethra in men and vagina in women are often painfully affected, the labia becoming excoriated. There is sometimes total suppression or retention of urine. After a few hours, convulsive motions often occur, consisting of tremors and twitches of the trunk, with severe cramps of the legs and arms. The general system now sympathizes acutely with the local mischief; there is a small, feeble, and rapid pulse, soon becoming imperceptible; coldness of surface, clammy sweats, lividity of hands and feet, palpitation, collapsed and anxious countenance, eyes red and sparkling, with severe conjunctivitis; tongue and mouth parched, and palate covered with small white ulcers. In the advanced stage delirium stupor and coma sometimes usher in the fatal event. In these cases the arsenic acts as an irritant, and the patient dies of inflammation; but the symptoms are not always uniformly marked, and it should be well considered that symptoms such as these do not alone constitute absolute evidence of the action of a poison, although they are highly suspicious and loudly call for a *post-mortem* examination, and a diligent search for arsenic.

3. When the patient is rescued from destruction after taking arsenic, or where death occurs after a protracted illness, the symptoms differ from those of the first and second class of cases; the *first train* of symptoms being those of violent but not fatal inflammation, and the *second train* those of nervous irritation. These generally come on when the former begin to recede, and they consist of *coma*, a peculiar *palsy of the arms or legs*, like that from lead poisoning, and anomalous symptoms of an *epileptic*, *tetanic*, or *hysteric* character, with or without *mania*. These cases (of the 3rd class) generally occur after taking a dose of arsenic consisting of a few grains only, or after the partial removal of a larger dose by an emetic. They are cases of great interest to the present inquiry, and we shall refer to them again.

On reviewing the above described phenomena, it appears that the primary effect of a large dose of arsenic in a state of solution is, a sudden depression of the action of the heart, under which it speedily ceases to beat; and the practical lesson it teaches us is, that where extreme prostration and frequent syncope occur after taking a large dose of arsenic, we may safely predict that the patient may live two hours, but cannot survive longer than about five or six. And as a corollary to this proposition, it may be said that these symptoms alone, when they thus terminate fatally in a few hours, offer a strong presumption that the patient has been poisoned by arsenic, even though there is as yet no evidence or even suspicion of his having swallowed the poison. It also appears from the second class of cases, that when the dose of the poison is not sufficiently strong to destroy the patient by arresting the action of the heart, its secondary effect is to excite acute and fatal inflammation of the alimentary canal, other mucous surfaces often partaking of the irritant action. By the third class of cases we are taught, that when the dose is not sufficiently strong to destroy the patient, either by its primary action on the heart, or by its secondary irritating effects on the stomach and bowels, the patient is then liable, after recovery from the inflammatory attack, to suffer severely and fatally from what may be called tertiary symptoms, consisting of lesions of the nervous system. As compared with the first and second class of cases, this last may be called *slow poisoning*; but whether it can be effected secretly, remains to be proved.

Before dismissing the subject of poisoning by large doses of the mineral, it may be as well to note that there are occasionally cases which appear to take an intermediate place between these different divisions. A patient may suffer severely at one and the same time, both from the depressing and the irritant effects of the poison: and there are cases in which he dies suddenly without any well-marked symptoms. So that we are bound to exercise caution, so as not, on the one hand, to refer every case of active inflammation of the alimentary canal, or of extreme depression of the heart's action, to the effects of poison; nor, on the other hand, to risk a strong opinion that the patient has not been poisoned, because some of the ordinary signs of arsenical toxication, or, indeed, *all* of them, are absent. The *post-mortem* examination, when it discovers palpable quantities of arsenic in the tissues, ought in every case to be conclusive and satisfactory in the present state of chemical science.

Upon the whole, then, it appears that the symptoms of arsenical poisoning in large doses, are, during life, *generally* so well-marked, that they can scarcely fail of exciting well-grounded suspicions.

We will now proceed to inquire into the action of arsenic in *very minute or medicinal doses.* This subject has been investigated with great care and attention of late years, and the results are highly interesting and satisfactory, though in some respects widely diverse from the conclusions which had been formed merely from observing the effects of the mineral in poisonous doses. It was natural enough to expect that the symptoms produced by minute doses would differ only in degree from those of the larger doses; and, strongly impressed with this conjecture, hundreds of medical men have repudiated its medicinal use altogether, although it has been known as a valuable medicine for more than a century. It has, however, been proved satisfactorily of late, that there is no foundation whatever for these misgivings; that the action of arsenic in minute doses is far from being similar in kind to its action in large doses—is, in fact, most dissimilar, and, in many respects, absolutely contrary, producing symptoms the very reverse of the toxical symptoms above described. Further, we are prepared to show that, properly administered and carefully watched, arsenic is a medicine remarkably safe, whether administered for a short time, or for months or years together,—that it never accumulates in the system, as some have believed; but that it is very rapidly eliminated,—that if it be continued for a long time neither does the system become so habituated to its action as to tolerate large doses, nor is any necessity created for persistence in its use, nor any corresponding danger incurred in suddenly relinquishing its habitual use. All this we are prepared to prove, not by allusions to newspaper stories, but by referring to our own experience, and that of our brethren at home, in its daily use; and then we shall be in some measure able to consider the merits of the ulterior question, the slow-poisoning process. We have said that the effects of minute medicinal doses are just the very opposite of those of the larger doses used for poisoning; the following table will show in what respects:—

| *In large doses, Arsenic* | *In medicinal doses, Arsenic* |
|---|---|
| Is almost always fatal. | Is never fatal. |
| Often produces extreme depression and collapse. | Is tonic and invigorating. |
| Impairs the action of the heart, oftentimes *ad deliquium*, exciting cold clammy perspiration. | Excites the action of the heart, and warms the extremities when they are otherwise disposed to be cold. |
| Excites nausea and vomiting. | Quickens the appetite and promotes digestion. |
| Irritates the bowels and excites inflammation, often with diarrhœa. | Never excites inflammation or diarrhœa; but sometimes restrains the undue action of the bowels. |
| Enfeebles and depresses the nervous system, sometimes producing stupor and coma. | Has a tendency to excite the nervous system, and is apt to make the patient restless at night. |
| Sometimes causes epilepsy. | Sometimes cures epilepsy, never causes it. |

The facts stated in the left-hand column of the above table require no proof. They are only too well known; for, unfortunately, poisoning by arsenic has been too frequent for its usual effects to be a matter of controversy. The contents of the right-hand column are not so universally known to be true. We shall therefore show on what grounds they are worthy of belief.

That arsenic administered with care, in proper medicinal doses, is never fatal, may be just as difficult to *prove* as any other negative, and yet there are reasons for believing it quite as satisfactory as demonstration could be. *First*, independently of experience, we might conclude from analogy that there can be no danger in a medicinal dose, inasmuch as this dose of arsenic is more remote from the poisonous dose than that of any other substance used in medicine. The safe dose of arsenic, and the largest generally required in medicine, is five minims of Fowler's Solution. Now, this is equivalent to one twenty-fourth of a grain of arsenious acid. But the smallest quantity of arsenious acid which has been known to be fatal is two grains, or about forty-eight or fifty times the quantity required for medicinal purposes. It appears, therefore, that it is a most unusually safe medicine; for let any other medicine, however harmless, be swallowed in like proportion, and it would certainly kill the patient. Take four grains of calomel, for example, as the medicinal dose; what if two hundred grains were swallowed at once? Or think of one hundred grains of opium, or thirty ounces of sulphate of magnesia, or thirty drachms of magnesia, or a quart or two of castor oil, or a drachm of extract of colchicum for a dose; or fifty wine-glassfuls of brandy! Who would not rather swallow two grains of arsenic than any one of these unheard-of doses? Most of the active materia medica would destroy life in four or six times their proper dose. Arsenic requires fifty times its proper dose, and then perhaps would rarely prove fatal. Surely we may infer that the proper dose of this medicine is a safe one. *Secondly*, the mild and gentle influence of arsenic when used as a medicine (similar, in many points, to that of quinine), ought to shield it from the imputation of being a dangerous medicine. If it ordinarily produces none of the symptoms of a large dose, even in the smallest degree, it is little less than absurd to expect it to kill! But, *thirdly*, lest the bugbear should still excite alarm, let us appeal to facts. Probably we have more distinct and extensive records of the therapeutics of arsenic than of any other article used in medicine. In the year 1848 the President and Council of the British (then called the Provincial) Medical and Surgical Association undertook an extensive inquiry among the members, as well as among other practitioners, as to their experience in the medicinal use of arsenic. The results of this important inquiry were published in the "Transactions" of the Association (vol. xvi., part 2); and in this "Memoir" is embodied the experience of seventy-five practitioners who had used arsenic (many of them largely, and some indiscreetly), and whose cases amount, in the aggregate, to several thousands. Yet, in their answers to special inquiries, it appeared that *not one of these practitioners had ever found the medicine either fatal or permanently detrimental to health.* We are tempted to ask, where shall we get such an amount of evidence as to the safety of any other medicine? But this is not all. We have ourselves pursued these inquiries for the last twelve years, far beyond the limits of this "Memoir;" and, including our own experience, we may say that we have come to the knowledge of about fifty thousand cases in which arsenic has been used as a medicine, not always discreetly, and as yet it does not appear to have been fatal in a single instance! *Rumours* of such poisonings have reached us, of course, but we have never been able to trace them home; nor have we the slightest hesitation in saying that no patient can ever perish in consequence of arsenical treatment, if it be discreetly conducted and the patient carefully watched. It is true that many writers on arsenic have cautioned us to watch for symptoms of gastritis, or of disturbance of the bowels, as signs of an overdose. Well, we *have* watched and waited for these symptoms for nearly forty years together, and we have no recollection of a single instance of enteritis, gastritis, or diarrhœa which we could trace to the use of arsenic. Indeed, we have notes of more than six thousand cases in which none of these symptoms are recorded, though we have often seen habitual diarrhœa restrained under its use, sometimes even to constipation.

Seeing, then, that large and poisonous doses of arsenic cannot be administered secretly, their action being violent and well known, and easily recognised, and seeing that minute doses if continued for years together, neither destroy life nor impair health, we are driven to the conclusion that if arsenic *can* be used as a slow and secret poison, it must be administered to the victim in doses neither very large nor very minute. And this brings us to the third branch of inquiry, namely—

What do we know of the effects on the human system of doses of arsenic of an intermediate strength, from a quarter of a grain or less to two or three grains, repeated at short intervals with a view to the destruction of life within a given period? And we shall divide our inquiry into two branches. 1. What are the symptoms during life? 2. What are the *post-mortem* proofs that the patient has died of arsenic?

It would seem reasonable to anticipate this inquiry by a confident conjecture, that the symptoms produced by doses of arsenic of intermediate strength, would naturally take an intermediate rank; that if the largest dose be poisonous and the smallest salutary, the medium dose may be expected to do neither good nor harm. We shall show, however, that this is not the case. It is true that our experience of these symptoms is extremely limited; but even this will be shown to be highly valuable, not only as regards the special object of this inquiry, but also in reference to the medicinal administration of arsenic. The subject may be best illustrated by quoting a series of cases, beginning with the smaller (intermediate) dose, and gradually ascending in the scale to the larger (intermediate) doses, making no allowance in the first instance for those exceptional cases in which the effects have been modified by idiosyncrasy.

The average medicinal dose of arsenic we have stated to be five minims of Fowler's Solution. On the testimony of the best authors most patients will bear this dose to be repeated twice or thrice a day for weeks together without inconvenience. Every ounce of this solution containing four grains of arsenite of potash, an ordinary dose of five minims represents the twenty-fourth of a grain of arsenious acid. In intermittents, however, the patient will bear doses of the twelfth of a grain, continued at intervals long enough to cure the ague. Few patients can, however, take this dose for a week without inconvenience. The conjunctivæ soon become inflamed, the eyelids swollen, the eyes smarting and watery. Let this dose be exceeded or doubled, and other symptoms appear, as we shall see.

*Case* I.—A clergyman of middle age had been taking for seven weeks for the cure of a leprous affection, fifteen minims of the liquor arsenici chloridi thrice a day, with benefit, but without any physiological sign. On applying to an ignorant druggist for a renewal of his medicine he was supplied with Fowler's solution in mistake. This is about three times as strong as the chloride, consequently he took three times a day about the eighth part of a grain (instead of the twenty-fourth) of arsenious acid in the form of arsenite of potash. At the end of a week he presented the following symptoms, which we copy from our case-book:—Conjunctivæ injected, the lower tarsal portion acutely inflamed, the eyelids and cheeks tumid and reddened. The soles of the feet and palms of the hands presented that kind of vesication known as the result of applying iodine ointment or tincture to the skin: darting pains in the head, inflammation and swelling of the knee, elbow, and finger joints, and a slight degree of pityriasis arsenicalis, with a mild form of diarrhœa. There was some degree of nervous agitation not amounting to trembling, *no sickness*, *no tingling of the hands.* We mention this latter circumstance because sickness and tingling of the hands were mentioned by several witnesses at the trial of Smethurst as pathognomonic of arsenical toxication in small and repeated doses. These symptoms soon subsided, and afterwards on a return of the disease (which had disappeared rapidly) he was able to take the usual doses of the chloride without inconvenience. That a perseverance in the large dose would ultimately have endangered his life, who can doubt? But here was an unusual amount of intolerance of arsenic.

Case 2 (related by the late Dr. Theophilus Thompson) :—" A gentleman to whom I had given Fowler's Solution for three months, persevered with eight-minim doses, after stiffness of the eyelids and nausea had occurred. Violent vomitings followed; perspirations and copious discharge of urine, which appeared to contain blood. In this instance some vesicles appeared on the feet (an appearance which I have somewhere seen mentioned as an occasional effect of arsenic) ; the eczema, which had been relieved, was now again aggravated, but after a suspension of the remedy for a few weeks, *it was resumed with more vigilance and precaution, and with ultimate recovery.*" Here the dose was not enormous, but it was long-continued, and might have been fatal at length.

Case 3.—Mr. Fox, of Falmouth, relates a case in which a physician most indiscreetly prescribed for an hysterical female, whose fits were sometimes of the epileptic character, the liq. potassæ arsenitis, in doses of *ten* drops, gradually increased to *twenty, four times a day*, or from one-twelfth to one-sixth of a grain of arsenite of potass. The throat became inflamed, she complained of severe burning pain in the stomach and bowels, inflamed conjunctivæ, and a papulous eruption. These symptoms were followed by an attack of mania, but how far it was due to the arsenic is not clear. She soon recovered from it, as well as from the symptoms of arsenical toxication.

Case 4.—In this case, which occurred under our own observation in the year 1838, a young lady took, by mistake, forty minims of Fowler's Solution (one-third of a grain of arsenious acid) thrice a day for six days—in the whole, six grains of arsenic! The symptoms recorded in our case-book were: Conjunctivitis, dimness of sight, giddiness, trembling of the limbs, restless nights, and other strange affections of the nervous system; but no sickness, diarrhœa, or griping, no tingling of the hands. She soon recovered from the immediate effects of the poison, but became intolerant of the smallest doses of medicine for years afterwards. This commencement of arsenic eating ill accords with the Styrian stories.

Case 5.—A well-marked case of (attempted) slow poisoning by arsenic is recorded by Flaudin (" Traité des Poisons, ou To[illegible]-logie," tom. 1, p. 510):—" A woman put daily into the soup of her fellow-servant a very small quantity of arsenious acid in powder, which was regularly rejected by vomiting after dinner, together with the food. This practice was continued at intervals for about six weeks, and the stomach became exceedingly irritable; there was also pain in the bowels, emaciation, spitting of blood, and such a degree of nervous irritability that a current of cold air excited convulsions. The patient now left the place, and passed two months in the country. Here she recovered her health, and returned to her usual occupations. The poisoner now renewed her attempts, and, to make sure, gave her victim one morning, in some coffee, a strong dose of arsenious acid in powder; violent vomiting ensued, and the poison was expelled with the food taken at breakfast; arsenic was detected in the vomited matter; and, under proper treatment, the woman recovered."

These cases, though differing from each other in some points, may be taken to represent the effects of arsenic in doses from one-eighth of a grain to one-third or more, repeated at intervals of a few hours. In each case, although there were present no symptoms of immediate danger, it cannot be doubted that slow poisoning had commenced; but the symptoms were so marked, that the process of poisoning, supposing the mineral had been administered in each case secretly with intent to poison, as happened in Case 5, must surely have been discovered had the poison been continued. Then, it may be observed that all the symptoms vanished rapidly when the poisoning process was suspended; and this teaches us another lesson as to the difficulty of secret and slow poisoning—namely, that should the murderer be interrupted in his course the victim may escape. And it also proves that the common opinion that the *remote* effects of small doses are dangerous, is utterly devoid of foundation. Patients recover from arsenic better than from mercury, iodine, antimony, or any other active medicine when administered in excessive doses.

We may now divide the cases into five distinct groups.

1. In doses of several drachms or ounces, partially or wholly dissolved, and not rejected by vomiting, we have excessive prostration, frequent and frightful fainting, and death within five (or at most six) hours.

2. In more common cases, where the quantity swallowed and dissolved is perhaps less than a drachm or two, we have heat and burning in the stomach, pain aggravated by pressure, frequent vomiting, and violent retching, and inflammation of the whole alimentary canal from the throat to the anus, with diarrhœa, tenesmus, strangury, and irritation of probably all the mucous membranes, then cramps and convulsions, cold sweats, and death in twenty-four hours.

3. When the poisonous dose is very small (from two grains to ten) we have *first* symptoms of inflammation of a less severe character, and *secondly*, coma, palsy, epileptic or tetanic symptoms with or without mania, death occurring after a long protracted struggle of several days, from which now and then a patient has been known to recover.

4. In doses from one-eighth to one-third of a grain, or more, we have quite a different set of symptoms, viz., conjunctivitis, dimness of sight, vesication of palms and soles, darting pains in the head, swelling and inflammation of the joints, pityriasis or lichen, giddiness, nervous agitation or trembling, sleepless nights, sometimes slight gastric irritation or diarrhœa, or, in the larger dose, vomiting. And these symptoms increase in severity in proportion as the course of poison is protracted, but rapidly disappear when the course is terminated. Some or all of these symptoms will be prominent in slow poisoning.

5. In medicinal doses (from gr. $\frac{1}{20}$ to $\frac{1}{40}$) we observe the effects to be never fatal, but generally tonic and invigorating, the extremities become warm, the appetite and the process of digestion quickened, and the patient becomes more active, and sometimes restless at night. A slight horizontal streak of sub-acute inflammation of that portion of the conjunctiva which lines the lower eyelid betokens the dose to be sufficient for all therapeutical purposes, and this dose may be continued, if needful, for several months or years without the slightest detriment to health, and it may be as safely arrested at any time.

To recur to group No. 4, in which we have the symptoms of slow poisoning, the *first thing* observable would be *the affection of the eyes*, which would be red and swollen and the conjunctivæ so inflamed and thickened as to produce partial blindness. The *second* symptom *vesication of the soles and palms*; the *third*, *nervous agitation* or *trembling*, *restless nights*, or horrid dreams. The *fourth*, *giddiness* and *darting pains in the head*. These four would generally be the leading symptoms; the casual or occasional would be swelling and inflammation of the joints, peeling of the cuticle, *i.e.* slight pityriasis or lichen; lastly, slight gastric irritation, perhaps diarrhœa or vomiting, chiefly when a larger dose is immediately rejected, what remains in the stomach exciting other symptoms of slow poisoning: neither tingling of the hands or enteritis ever occur. These symptoms may be expected to ebb and flow or vary in severity if the poisoner fails in his purpose of giving his victim a constant succession of doses, and if he should at last succeed in administering a larger dose than usual, then, unless it were immediately rejected by vomiting, fatal inflammatory symptoms would probably close the tragedy. We ask, now, would not these symptoms excite suspicion and attention, more especially if they were better known and more carefully committed to memory by medical practitioners?

*Secondly,* the *post-mortem* examination, supposing the murderer succeeded, would generally reveal the truth. There would be, in the vast majority of cases, more than traces of arsenic in the tissues and in the blood, and this latter would probably have lost its coagulating power; but supposing the sufferer survived for a fortnight or more after the last dose of arsenic, then the whole of the poison might be eliminated before death, so at least the following case would seem to prove:—A gentleman had been taking arsenic in medicinal doses (four drops of Fowler's Solution thrice a day) for the cure of a cutaneous disease. The course had been protracted for twelve months or more, when he died of a well-marked attack of typhoid fever then endemic in the neighbourhood. He had taken no arsenic for about a fortnight before he died. One of his medical attendants ignorantly persisted in the opinion that the arsenic had poisoned him; and at the *post-mortem* examination took away with him a portion of the liver, some blood, and the fluid contents of the stomach and bowels. However, decomposition was rapid and not a trace of arsenic was found.* It is not therefore impossible, even in a case of actual poisoning by arsenic, that the whole of the poison might be eliminated if the patient survived for two or three weeks the last dose of arsenic. This would be a most puzzling case for a jury; still until the symptoms of slow poisoning are better known to the profession, a verdict of murder on this evidence of symptoms alone would rarely be justifiable. If, however, arsenic be detected in the food or drink offered to the victim, that would materially alter the case.

The common belief then, that arsenic in small doses *accumulates* in the system, is altogether erroneous. It is true that arsenic in medicinal doses is what is called a cumulative poison; *i.e.*, its effects accumulate because it acts very slowly. The second or third week of a course of this medicine often shows symptoms of an overdose, which did not appear during the first week, so that the dose must be *decreased.* This is peculiarly true if its action on the vascular system is seen in the conjunctivæ. When it acts primarily on the nervous system, exciting the muscular irritability, and disturbing the rest at night, these symptoms gradually subside, while the vascular irritation increases. And this is so uniformly the case, that it is impossible to con-

---

* MM. Danger and Flaudin found no trace of arsenic in the bodies of animals to which doses of fifteen grains daily had been given; the bodies were examined three days after the last dose.

ceive that the Styrian peasants can go on constantly increasing the dose, and finding its inflammatory effects less and less obvious. There *are* exceptions to all rules, but the actual habits of a country or nation are not the exception but the rule. Therefore, when we are told even by a medical journalist that the arsenic eaters "begin quietly with a bit of the size of a millet seed and gradually arrive at a lump of the size of a pea, weighing from four and a half to five and a half grains, daily," we simply reply *we* cannot accept the statement; and we feel a little ashamed that such things should be soberly and seriously related in a medical periodical.

The cumulative effects of even medicinal doses of arsenic, when administered rashly in *constantly increasing* doses, without waiting a week or two to observe their effect, might prove very serious in the case of a patient who, from idiosyncrasy, happened to be peculiarly susceptible of the toxical influence of the mineral. Formerly this was the common practice, and it often produced alarming symptoms, but we believe never a fatal result. It is indeed to this peculiar idiosyncrasy chiefly that we should be disposed to attribute the injurious effects of arsenical papers in the apartments of dwelling-houses, as also the sufferings of, here and there, a handler of the arsenical preparations used in imparting a green colour to the flowers worn on ladies' bonnets. The cry of universal injury received from these sources we believe to be exaggerated. Nevertheless, we are strongly of opinion that the use of arsenical greens, for mere purposes of ornament, ought to be strictly forbidden by law. If only the life of one in a thousand were endangered, this would be too great a sacrifice for the purpose of mere ornament. And it surely becomes every medical practitioner to use the whole weight of his influence in this direction. If our lady sanitarians have shown a little more of zeal and philanthropy, than of science and judgment, in their urgent expostulations, we of the profession have certainly shown, as a body, no great excess of zeal in this matter. And the reason is we have taken too little pains in examining the physiological merits of the case. We have not even appreciated justly the difficulties of the subject. And yet this is the first step towards understanding it. We must not be content with listening incredulously to idle and contradictory tales. Standing, as we do, at the bar of public opinion, as witnesses supposed to be well-informed, and often appealed to as to the truth of the public reports on such subjects, it is surely no credit to us either to *appear* indifferent or to *be* ignorant of anything which concerns the sanitary welfare of the community. Most of us have much to learn, and happily there is here and there one who seems determined to investigate the matter. The recent ingenious and highly interesting and suggestive researches on Slow or Chronic Arsenical Poisoning, by Dr. Harley, Professor of Medical Jurisprudence in University College, may be cited in illustration. These researches consisted of a lengthened series of experiments on animals. The poison was injected into the veins, and while some of the experiments lasted only twenty-five minutes, others extended over a period of eighty days. From these experiments it would appear that the effects of arsenic on the digestive canal differ in the acute and chronic form of poisoning. So different, indeed, are the morbid changes presented in the two cases, that the effects of the one could never be deduced from those of the other. The more chronic the action of the poison, the more uniformly are its effects distributed over the digestive canal; whereas in the acute form of poisoning, the effects of the toxic agent are more especially observed in the stomach. From certain researches which Dr. Harley also made upon the direct action of the poison on the blood, it would seem that arsenic has the power of retarding the metamorphosis of the constituents of that fluid, and thereby affecting the tissue change. The conclusions which Dr. Harley has derived from the whole series of his experiments he has thus summed up:—

1st. That arsenic has a specific action on the digestive canal.

2nd. That the action of arsenic on the digestive canal is manifested irrespectively of its mode of administration.

3rd. That the direct contact-action of arsenic with the mucous membrane is slight in comparison to the influence it exerts through the blood.

4th. That the symptoms manifested during life, as well as the morbid changes found after death, differ very materially in the acute and chronic forms of poisoning.

5th. That whereas in the acute form of poisoning the morbid changes are most marked at the cardiac end of the stomach, in the chronic form they are most visible towards the pyloric extremity.

6th. The more gradual the poisoning, the more manifest is the action of the poison on the intestines, and the less visible are its effects on the stomach.

7th. Death may occur from arsenic so rapidly that no apparent structural change has time to occur.

8th. That the immunity from symptoms of poisoning enjoyed by arsenic-eaters most probably arises from their taking the substance in a solid form, and consequently that but a very small portion of what is swallowed enters the circulation.

9th. That the beneficial effects of small doses of arsenic are due to the power it possesses of diminishing tissue-change by its peculiar action on the blood.

10th. That the prejudicial effects of arsenic, when taken in excess, are due to its destroying the property possessed by the constituents of the blood of combining with oxygen, and thereby becoming fitted for the purposes of nutrition.

We have cited Dr. Harley's researches on account of their suggestiveness, and more especially for the important hints they furnish for the future study of chronic arsenical poisoning in man. Let us encourage such valuable labours, and let us endeavour to discover the real causes of any discrepancy of opinion which may prevail amongst ourselves

The question of *arsenic eating*, which, at first sight, seems to ignore all the supposed dangers arising from the commercial use of arsenic, is easily disposed of. *There is no reliable evidence whatever that the system can acquire a tolerance of arsenic in doses which would prove poisonous to the generality of adults.* The asserted examples of individuals having become arsenic-proof are very rare, and in these instances it is stated that the poison was taken in the solid, crystallized form. Assuming the correctness of these statements, it is not improbable, as Dr. Harley suggests (a suggestion which accords entirely with our own opinion) that but a very small portion of what is swallowed enters the circulation. If the arsenic-eater happened to have an acid in the stomach, a smooth piece of arsenious acid would prove nearly insoluble, and might pass through the alvine canal all but unchanged. In this manner it is not impossible that a certain *accidental* immunity from the toxical effects of the poison might, from time to time, be observed; and even that the minute portion which it is to be assumed would be dissolved and would pass into the blood, might have exercised a medicinal effect, invigorating the appetite for the time, and tending to give plumpness and strength. But that examples of apparent tolerance of arsenic in poisonous doses are most rare, is the only safe conclusion which can be arrived at from the exceedingly unsatisfactory accounts we possess of the asserted arsenic-eaters of Styria. On the other hand it is important to note that *arsenical poisoning* (not necessarily criminal) is very common in Styria. This fact, while it indicates the familiarity of the peasantry with the poison, and furnishes indirect confirmation of the stories of the frequent use of arsenic by them, shows at the same time that the poison has not lost its deleterious properties by change of locality, and justifies the very gravest doubts of the assertions respecting arsenic eating.

Assuming, however, the correctness of these stories, it is impossible, until we have far different evidence than that which we now possess, to look upon instances of arsenic-proof individuals otherwise than as rare exceptions rather than the rule, and that their immunity is an *accidental circumstance* rather than an acquired habit.

Notwithstanding the period during which the story of the Styrian toxophagists has been patent, we are still without those precise details which would enable us to form a just scientific judgment of its merit; but of this there can be no doubt, that the popular versions of the story are entirely wanting in credibility.

---

